# Oral Bioavailability Enhancement of Raloxifene with Nanostructured Lipid Carriers

**DOI:** 10.3390/nano10061085

**Published:** 2020-05-31

**Authors:** Aditya Murthy, Punna Rao Ravi, Himanshu Kathuria, Shrinivas Malekar

**Affiliations:** 1 Differentiated Formulations, Strides Pharma Science Ltd., R & D Centre, J.P. Nagar 2nd Phase, Bangalore 560083, Karnataka, India; adityamurthy1212@gmail.com; 2 Department of Pharmacy, BITS-Pilani Hyderabad Campus, Hyderabad 500078, Telangana, India; himanshukathuria01@u.nus.edu (H.K.); malekarshrinivas@gmail.com (S.M.); 3 Department of Pharmacy, National University of Singapore, 18 Science Drive 4, Singapore 117543, Singapore

**Keywords:** solid lipid nanoparticle, nanostructured lipid carriers, bioavailability, glyceryl behenate, raloxifene, osteoporosis

## Abstract

Raloxifene hydrochloride (RLX) shows poor bioavailability (<2%), high inter-patient variability and extensive gut metabolism (>90%). The objective of this study was to develop nanostructured lipid carriers (NLCs) for RLX to enhance its bioavailability. The NLC formulations were produced with glyceryl tribehenate and oleic acid. The particle characteristics, entrapment efficiency (EE), differential scanning calorimetry (DSC), in vitro drug release, oral bioavailability (in rats) and stability studies were performed. The optimized nanoparticles were 120 ± 3 nm in size with positive zeta potential (14.4 ± 0.5 mV); % EE was over 90% with the drug loading of 5%. The RLX exists in an amorphous form in the lipid matrix. The optimized RLX-NLC formulation showed sustained release in vitro. The RLX-NLC significantly (*p* < 0.05) enhanced oral bioavailability 3.19-fold as compared to RLX-free suspension in female Wistar rats. The RLX-NLC can potentially enhance the oral bioavailability of RLX. It can also improve the storage stability.

## 1. Introduction

Osteoporosis is a disease condition with a greater risk of fracture as a cause of decrease in bone mineral density and bone quality [1]. There are many reported causes for osteoporosis, but menopause is the most common and potential cause. Post-menopause, levels of the estrogen fall which leads to disturbed density of bone minerals with susceptibility to bone fractures [2]. It affects women worldwide including in Europe, USA and Japan [3,4]. Raloxifene hydrochloride (RLX) is a popular selective estrogen receptor modulator [5], which is used to prevent and treat postmenopausal osteoporosis. It has proven effective to preserve bone density, preventing the incidence of vertebral fractures in postmenopausal women [5,6]. It is also used for the treatment of breast cancer [5,7]. According to the Biopharmaceutics classification system and the Biopharmaceutics drug disposition classification system, RLX is Class II drug [8]. RLX is highly permeable and absorbed 60% by the oral route; however, its bioavailability is only 2% in humans as a cause of extensive pre-systemic intestinal glucuronidation and permeability glycoprotein (Pgp) efflux [9,10]. The bioavailability of RLX in animals varies with species from 0–39% [11].

The oral route is the most preferred route for the administration of drugs as it offers the greatest patient compliance. It accounts for more than a 50% share of the global drug delivery market [12]. The various approaches for the oral bioavailability enhancement of RLX, such as dry suspensions of cyclodextrin inclusion complexes [13], mesoporous carbon nanospheres [14], proliposome powders [15], solid dispersions [16,17] and self-microemulsifying drug delivery system solids [18] have been explored previously. Lipid nanocarriers can be suitable nanocarriers for RLX owing to their capability to mainly deliver poorly soluble and lipophilic drugs [19]. Lipid nanocarriers have also been shown to decrease inter-individual variability [20]. The orally administered lipid nanoparticles enter into systemic circulation by different uptake mechanisms such as receptor-mediated endocytosis, non-specific transcellular transport, paracellular transport and M-cell-mediated transport [21]. The enhanced oral bioavailability of drugs loaded in solid lipid nanoparticles (SLNs) is due to their small particle size, increased surface area and surface properties (like charge) that improve their bioadhesion to the gut wall, leading to prolonged residence time in the gastrointestinal tract (GIT). Moreover, the production of the mono and diacylglycerols during the digestion of triglycerides by lipases in the GIT induces the secretion of bile salts that together with mon and diacylglycerols form micelles. These micelles further help to enhance the oral absorption of drugs. Moreover, SLNs can also facilitate lymphatic transport by initiating the formation of lipoprotein and intestinal lipid flux giving the bulk bioavailability of drugs [22]. The composition of SLNs and its excipients such as the amount of surfactant can significantly change the particle size, which also affects the lymphatic transport. The direct uptake of lipid nanocarriers through M-cells and lymphatic transport also plays the major role in the enhancement of their bioavailability [23].

In recent years, lipid nanocarriers have been shown to enhance the oral bioavailability of RLX [11,17,24,25,26]. Nanostructured lipid carriers (NLCs) are the modification of solid lipid nanoparticles (SLNs) in which part of the solid is replaced by another solid lipid or an oil [27]. The limitations of SLN-like drug expulsion during storage and low entrapment efficiency were studied by the researcher leading to the development of a second generation of SLNs as NLCs [27,28]. It was shown to have better physical stability than SLNs by reducing the expulsion of the drug during storage. Replacing the part of the solid lipid by an oil causes an imperfection in the lattice structure of solid lipids. The increased imperfections in the solid lipid lessen the chance of drug expulsion during storage while retaining the loading capacity; hence, this improves formulation stability as compared to SLNs [27,29]. Recently, Puro et al. reported the transdermal delivery of raloxifene using NLCs, which were later loaded into gel for topical application [30]. Shah NV et al. described RLX-loaded NLCs based on glyceryl monosterate, formulated using the solvent diffusion method, which involves multiple steps in addition to the use of toxic organic solvents [31]. In contrast, the hot homogenization method involves a lesser number of steps and minimal factors to optimize the formulation, which can be easily controlled. Moreover, the hot homogenization can eliminate the use of toxic organic solvents; hence, avoiding potential safety hazards.

In this study, we formulated the RLX-loaded NLCs to enhance its oral bioavailability and improved stability. The RLX-NLCs were prepared with glyceryl behenate (solid lipid) and oleic acid (liquid lipid) similarly to a previously reported method from our group for the preparation of SLNs [11,32]. The optimization was done based on factors such as surfactant concentration, sonication time and lipid amount. The particle characteristics (size, zeta potential and polydispersity index (PDI)) and entrapment efficiency were taken as the responses to optimize the formulation. The optimized formulation was carried forwards for its in vitro release, then the oral bioavailability studies in female Wistar rats to prove its potential in bioavailability enhancement and finally the formulation stability.

## 2. Materials and Methods

### 2.1. Materials

The raloxifene hydrochloride was obtained as a gift sample from Apotex Research Pvt. Ltd., Bangalore, India. The high purity glyceryl tribehenate (C_69_H_134_O_6_, molecular weight 1059.8 g/mol) was purchased from M/s Himedia Pvt. Ltd. (Hyderabad, India). The Kolliphor P407 (poloxamer 407) (P407) and mannitol were procured form Signet Chemicals, Mumbai, India. The oleic acid was procured from SD fine chemicals Ltd., Mumbai. All the other chemicals used were of analytical grade and the solvents were of high performance liquid chromatography (HPLC) grade. Freshly collected Milli-Q water (Millipore, Billerica, MA, USA) was used in the formulation preparation and the preparation of the aqueous mobile phase of the HPLC analysis.

### 2.2. Preparation of NLC

All the formulations were prepared by a hot homogenization process followed by ultra-sonication. Firstly, glyceryl behenate and oleic acid were melted in a 100 mL glass beaker maintained at a temperature of 80 °C ± 4 °C using a silicone bath with a magnetic stirrer. The drug was added with the melted lipid–oil mixture and stirred for uniform mixing. The aqueous phase consisted of P407 dissolved in the cold water using a magnetic stirrer (Model: 1MLH, REMI Labworld, Mumbai, India) at 1000 rpm. The aqueous phase was maintained at the same temperature as that of the molten lipid phase to prevent recrystallization during stirring. The hot aqueous phase was added drop wise into the oil phase with continuous stirring at 1000 rpm for 5 min to form pre-emulsion. It was then homogenized using a high shear homogenizer (Polytron PT 3100D, Kinematica, Lucerne, Switzerland) at 10,000 rpm for 5 min and subjected to sonication using a probe Sonicator (Vibra cell, Sonics, Newtown, CT, USA) on pulse mode (20 s sonication 10 s off cycle; attached with ¾” solid tapered tip that could vibrate up to 120 µm amplitude at 100%) for varied time intervals (Table 1) to form nanoemulsions. Subsequently, the nanoemulsions was rapidly cooled down in an ice bath to form NLCs and finally diluted up to 50 mL by the addition of 20 mL cold water (1–2 °C) and incubating that in an ice bath for 15 min. The final NLC dispersions were stored at 4 °C and at room temperature for further analyses.

### 2.3. Formulation Optimization

Firstly, the method of preparation was optimized followed by the formulation optimization which was done by keeping the phase ratio constant; the effects of magnetic stirring, homogenization and sonication were observed individually and in combination. Secondly, for optimizing the NLC formulation, various parameters were studied sequentially. It was done by varying factors in the following sequence: the amount of oleic acid, the surfactant concentration and then the sonication time. The P407 concentration referred to the % P407 used to make the formulation, while the final P407 concentration after dilution with water was lesser than that shown in Table 1. Herein, the % P407 in the final formulation was 1%, 2% and 3%. The particle characteristics like the particle size (Z-average, d50, d90, d95), PDI, zeta potential and the formulation characteristics like the % EE and the percentage of cumulative release (% CR) were measured as responses (See Table 1).

### 2.4. Assay Method

A total of 200 µL of the formulation was added to 800 µL of chloroform to completely dissolve the lipid part. Then, the above mixture was evaporated to remove all the volatile content like water and chloroform by placing it in a hot air oven for 30 min at 60 °C. Subsequently, 10 mL of methanol was added into the dried mixture for the reconstitution of RLX. The glyceryl behenate was insoluble in methanol while the drug was freely soluble. The methanolic solution of the drug was analysed in HPLC (Model LC-20AD, Prominence Liquid Chromatograph, Shimadzu Corporation, Kyoto, Japan) using a validated method after suitable dilution [33].

### 2.5. Entrapment Efficiency

The free drug content was measured by the dialysis bag method. The dialysis method was chosen after unsuccessful attempts to separate the free drug and the nanoparticles using a centrifugation method. An amount of 5 mL formulation was taken into the dialysis bag (Spectra por 2, Fisher scientific, Waltham, MA, USA) with molecular weight cut off of 12–14 kDa, and kept in 140 mL dialysis media (pH 7.4 phosphate buffer and polyethylene glycol 400 (PEG-400) in the ratio of 4:1) under 150 rpm in a modified type I dissolution apparatus for a maximum of 3 h. This method was validated by spiking the free drug solution into a placebo NLC formulation and ensuring over 95% drug recovery in a 2 h period. The media volume was 140 mL, ensuring that the sink condition was maintained, whereas the drug concentration in the release media was not more than 35 µg/mL, which was nearly 10 times higher than that of the drug solubility i.e., 300 µg/mL. The samples were collected at 1, 2 and 3 h to ensure the reliability of the method for each measurement, and were later analysed in HPLC using a validated method.

The percentage entrapment efficiency (% EE) was calculated using the formula mentioned in Equation (1):

(1) $$\mathrm{Percentage}\;\mathrm{EE}\;\left(\%\;\mathrm{EE}\right)=\frac{X_{total}-X_{free}}{X_{total}}\times 100$$

where $X_{total}$: total amount of RLX loading obtained using the formulation assay; $X_{free}$: free RLX in the media obtained using the dialysis method.

### 2.6. Particle Size and Zeta Potential

The particle size was measured using dynamic light scattering and the zeta potential was measured using electrophoretic light scattering. The particle size was calculated as the particle size diameter (Z-average, d50, d90, d95) of the main population and the PDI was calculated as a measure of the width of the particle size distribution using a Malvern Zetasizer NANO ZS (ver. 6.01 MALVER Instrument Ltd., Worcestershire, UK). The formulation samples were analysed for the particle size and zeta potential in the automatic run mode, which allowed for repeated measurements with accuracy. The measurements were run at 25 °C with 2 min of equilibration time. The zeta potential of the formulations was measured using the same equipment with a folded capillary zeta cell. All the measurements were done in triplicate.

### 2.7. Differential Scanning Calorimetry

The differential scanning calorimetry (DSC) was done for the RLX, glyceryl behenate, P407 and the melt dispersion of glyceryl behenate with RLX and oleic acid. The melting dispersion was prepared by dissolving the RLX in the melting of glyceryl behenate with oleic acid (10 and 30% *w/w*). The thermal analysis was performed using a DSC 60 (SHIMADZU Corporation, Kyoto, Japan). Firstly, the heating chamber was equilibrated at 35 °C and then 2 mg samples were accurately weighted and sealed in an aluminium pan. The sealed aluminium (with the sample) pan was placed on sample plate and an empty sealed aluminium pan on the other plate as a reference. The nitrogen gas flow of 30 mL/min was used to purge the system; the heating temperature was in the range 35–300 °C at a heating rate of 10 °C/min.

### 2.8. In Vitro Drug Release

The release study was done using the dialysis bag method. Here, 2 mL formulation was taken into the dialysis bag. The dialysis membrane was put into the 140 mL of media (0.1 N HCl (2 h) and pH 6.8 phosphate buffer for up to 12 h) in a 250 mL beaker at 37 °C ± 0.5 °C with continuous stirring at 100 rpm. An amount of 0.1% *v/v* Tween was also added to the media to ensure the sink condition. Then, 140 mL media volume was taken to ensure that the sink condition was maintained, and even if the whole drug would dissolve in the media, then it would produce a concentration of no more than 14 µg/mL, which was more than 20 times that of our drug solubility. At various time points (0.083, 0.25, 0.5, 1, 1.5, 2, 3, 4, 6, 8, 12 h), 2 mL samples were collected and an equal volume of the media was replaced. All the samples were analysed in HPLC using a validated method.

### 2.9. Stability Studies

The accelerated stability studies were carried out for the optimized RLX-NLC as per the guidelines of the International Conference on Harmonisation Q1A (R2) (2003). The formulation was stored in three 50 mL transparent tarson tubes in a stability chamber maintained at 25 °C ± 2 °C/60% ± 5% relative humidity (RH). It was subjected to a stability test over a 6-month period for particle size, PDI, zeta potential and % EE with sampling times of 1 month, 3 months and 6 months. Likewise, 3 tubes were kept in cold conditions at 4 °C and the stability was measured for 6 months. The reduction in the entrapment efficiency over time could affect the bioavailability enhancement, which may compromise the therapeutic effect of the formulation. The changes in the entrapment efficiency over 6 months was measured to observe the stability of the formulation, which can ensure that the effectiveness of the NLC formulation remains the same over time.

### 2.10. In Vivo Pharmacokinetics

The experiment was carried out in female Wistar rats which were fasted overnight for 12 h with access to water only. They were acclimatized to the environment one day before the experiment. The rats were divided into two groups of 3 each for the control (RLX suspension) and the test (RLX-NLC, NLC 3) groups. The two groups of rats were housed separately in propylene cages (38 cm × 23 cm × 10 cm under laboratory conditions of a controlled environment at a temperature 25 °C ± 2 °C and 60% ± 5% RH. All the animals were dosed at 00:30 p.m. and the RLX was suspended in water using methylcellulose to give a concentration of 1 mg/mL. The volume of administration was 15 mL/kg and the doses were 15 mg/kg in all the control group animals. The formulations were administered orally with the aid of a syringe and a rat oral feeding needle. The blood samples were drawn by retro-orbital venous plexus puncture with the aid of bleeding capillary tubes. The blood samples were centrifuged at 3400 rpm for 10 min into separate plasma. The supernatant (plasma) was collected and stored at −80 °C until analysis.

The samples were taken according to the regulatory guidelines. The rats were anesthetized in an anesthetic chamber using diethyl ether each time before the collection of the blood samples. At various time intervals ~250 µL of blood was collected in 0.5 mL Eppendorf tubes containing 25 µL anticoagulant (3.4% w/v sodium citrate). A validated HPLC method was used for the analysis. The RLX detection was performed at 289 nm, using an ZORBAX SB-C8 (5 µm, 4.6 × 150 mm) (Agilent Technologies Inc., Santa Clara, CA, USA) analytical column. The mobile phase composition was 63:37 (*v/v*) of pH 4.5 20 mM ammonium acetate buffer (adjusted with acetic acid)/acetonitrile at a flow rate of 1.0 mL/min. The plasma samples (0.1 mL) were transferred to 1.5 mL Eppendorf followed by the addition of 150 µL acetonitrile and 50 µL mobile phase premix to precipitate the plasma proteins. The above mixture was vortexed for 5 min and centrifuged at 10,000 rpm for 10 min. Then, 50 µL of the supernatant was injected into the column. The calibration curves for the RLX in plasma were drawn every time during the analysis using the lower quantifying concentration 150 ng/mL, the middle quantifying concentration 600 ng/mL and the higher quantifying concentration 1300 ng/mL [11,33].

## 3. Results and Discussion

### 3.1. Particle Size and Polydispersity Index

**Figure 1:**
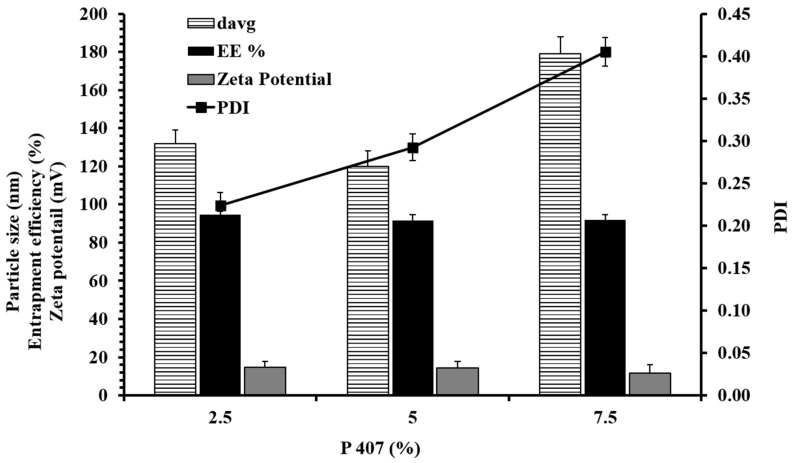
Effect of surfactant concentration on particle characteristics (d-avg, PDI, zeta potential) and percentage of entrapment efficiency (% EE).

The size of the nano formulations is an important factor that affects the stability, lymphatic transport and bioavailability [34,35,36,37]. Li et al. had reported that the lowest size NLC (100 nm) in their study was the most stable. Though all NLC sizes (100, 200 and 300 nm) could rapidly penetrate the duodenum versus the later part of the intestine (jejunum, ileum and colon), however, smaller NLCs (100 nm) showed the highest pharmacokinetics parameters (C_max_ and Area under the curve (AUC)). Herein, the particle size of the NLCs is presented as the Z-average, d50, d90 and d95. The Z-average depicts the mean hydrodynamic diameter of the particles. The other diameters in this study (d50, d90 and d95) depict the percentage of undersized particles. Table 2 presents the particle characteristics of the NLC formulations and Figure 1 shows the effect of the P407 concentration on the formulation characteristics. It revealed that the formulation composition and the process variables like the surfactant concentration, homogenization, sonication and the amount of oleic acid significantly influenced the particle sizes. Introducing the homogenization step for 3 min in addition to the magnetic stirring significantly reduced the average particle size of NLC. The homogenization helped in reducing the particle size and making the pre-emulsion more homogeneous. The homogenization alone was not enough to get the further reduction in particle size and PDI. It was possible to achieve only by increasing the time of homogenization and the homogenization rpm or else by the use of ultrasonication. However, the homogenization parameters i.e., the time and rpm, were difficult to manage because of the foam generation during homogenization.

The extensive foam generation during the homogenization was observed at a higher rpm, which lead to larger visible particles and aggregates floating in the formulation. Therefore, the homogenization followed by sonication was used instead of only the sonication or homogenization. The ultrasonication process was used for a further size reduction and to reduce the PDI. Surajit Das et al. have shown that an increase in sonication time caused a decrease in the particle size to a certain extent [38]. However, after a certain time of sonication, the particle size did not change drastically. Herein, we studied the effect of sonication time for 10 min and 20 min for the optimized formulation. There was no significant difference in the particle size between the NLCs formulated with 10 min sonication and those with 20 min sonication. However, a slight increase in the particle size was observed with a higher sonication time. This can be attributed to the water loss as a cause of evaporation due to the heat generation during sonication for 20 min. Furthermore, an increment in surfactant concentration during the formulation as a cause of evaporation can also affect the particles characteristics. Therefore, we suggest that a longer sonication time should be avoided. The smaller size (100 nm or less) NLCs could also be produced by the use of other methods such as solvent diffusion [31,36], modifying the composition [31] and the use of microfluidic devices [39].

The particle size was found to decrease when the oleic acid amount varied between 9.1% and 30% to replace a part of the glyceryl behenate (solid lipid) amount. The decrease in particle size with an increase in oleic acid could be attributed to the reduction in the interfacial tension in the presence of oleic acid. Uprit, S. et al. have previously shown that the amount oleic acid in the NLCs can have an impact on the viscosity and interfacial tension, because of which it can form smaller particles [40]. The P407 concentration was also found to affect the particle size at a higher concentration. There was a significant increase in the particle size when the P407 concentration was increased from 2.5% to 7.5% during the pre-emulsion formation. Sanjula B. et al. have also reported that an increase in poloxamer concentration in SLNs can reduce the lymphatic uptake as a cause of an increase in particle size [23]. The increase in particle size can be attributed to the aggregation of particles at a higher P407 concentration.

**Figure 2:**
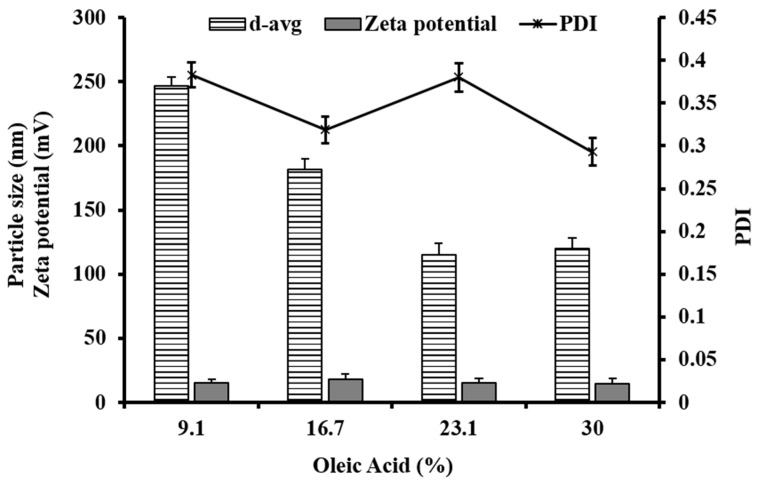
Effect of the amount of oleic acid (% *w/w*) on the NLC particle characteristics (d-avg, zeta potential and PDI).

The PDI is also an important parameter to interpret the distribution of particle size. It was utilized to optimize the NLC formulation and for interpreting the storage stability of NLCs. For the PDI system from 0 to 1, the PDI close to 0 shows a monodisperse dispersion while the value 1 shows a wide distribution. The PDI < 0.2 is considered as a narrow size distribution for nano formulations. The results showed that the PDI can be affected by homogenization rpm, time of homogenization and sonication, method of formulation, temperature, etc. The homogenization step incorporated before ultrasonication significantly reduced the PDI as compared to the NLCs prepared without homogenization. The PDI was found to increase with the increase in sonication time from 10 to 20 min. This can be attributed to changes occurring during the NLC preparation like an increment in the concentration of the surfactant as a cause of water evaporation and heat generation leading to changes in the distribution of RLX in the aqueous and lipidic phase. The increase in surfactant concentration also increased the PDI of the NLCs. There was no direct correlation between the PDI and the percentage of oleic acid (see Figure 1 and Figure 2). Hence, considering the particle sizes and the PDI, the optimum NLC formulation was NLC 3, which was carried forward for stability and pharmacokinetics studies.

### 3.2. Zeta Potential

The zeta potential represents the charge of the particles. It indicates the degree of repulsion between similarly charged particles in the dispersion. This repulsion force helps in predicting the physical stability of the formulation [41,42]. Therefore, the zeta potential was a useful parameter for optimizing the formulation. The RLX suspension in water showed +25 mV zeta potential while the placebo formulation showed −13.7 mV and all the other NLCs also showed positive zeta potential. The negative zeta potential in the placebo could be attributed to the surface-ionized oleic acid and the positive zeta potential in the formulation can be attributed to the drug-enriched shell of RLX. This phenomenon of a drug-enriched shell has been reported previously. The core of the lipid nanocarriers forms when the temperature of the dispersion is reduced to the recrystallization temperature of the lipid, and due to this the drug concentrates in the still liquid outer shell of the SLN [38,42,43]. Therefore, the drug-enriched outer shell and zwitterion nature of RLX support the positive charge of the particles.

The results showed that (see Figure 2) there was no significant change in the zeta potential with the change of surfactant concentration from 2.5% to 7.5%. However, a slight decrease (not significant) in the zeta potential in the 7.5% NLCs as compared to the 2.5% and 5% NLCs was observed. This can also be correlated to the higher particle size (see Figure 2) in the 7.5% NLCs than the 2.5% and 5% NLCs. There was no specific correlation between the zeta potential of the NLCs in relation to the sonication time and the oleic acid content.

### 3.3. Entrapment Efficiency

There was no significant change in the % EE with the change in the surfactant concentration (see Table 3). This can be attributed to the efficient drug loading with enough amount of lipid, which enables the available RLX to be mostly loaded into the NLCs. The results showed that the % EE of the NLCs was not affected by changing the sonication time from 10 to 20 min, while increasing the P407 concentration reduced the % EE of RLX (Table 3). The reduction in the % EE was obvious as the drug-apparent solubility increases with the increase in the surfactant concentration. However, the optimum amount of surfactant is essential as it helps in getting smaller-size NLCs and provides stability while also retaining good entrapment efficiency. Pandita et al. have shown that poloxamer 188 concentration beyond 1.5% w/v decreased the % EE of paclitaxel, while 0.5 % to 1.5% w/v surfactant increased the % EE [44]. In contrast, Pezeshki et al. showed an increase in the % EE of Vitamin A palmitate with the increase in % P407; however, the stability of the NLCs was reduced with a higher surfactant concentration [45].

### 3.4. In Vitro Drug Release

The NLCs prepared with different surfactant concentrations were analyzed for cumulative drug release to measure the amount of RLX release in the GIT pH conditions. The % CR of RLX was found to be the lowest for the formulation made with 2.5 % surfactant and highest for the formulation made with 7.5% surfactant (Table 3).

**Figure 3:**
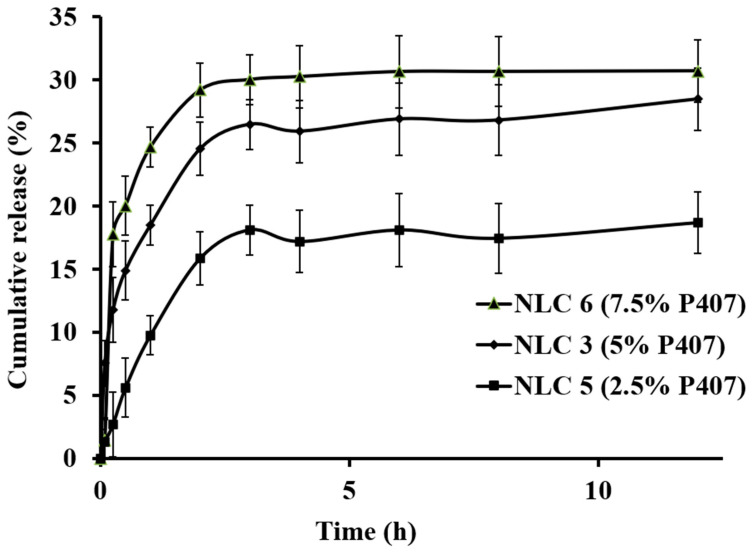
Effect of the P407 concentration on the percentage cumulative release of RLX from the NLCs.

All the tested formulations show bi-phasic release, where the initial burst release of RLX can be seen until 1 h followed by slow release. The bi-phasic release of drugs from the SLNs and NLCs prepared using hot homogenization has also been reported earlier [11,46]. The initial burst release can be attributed to the dissolution of both the free RLX and RLX release from the drug-enriched outer shell. The second phase i.e., the slow release, is attributed to the core of the NLCs. The % CR of RLX from the NLCs was no more than 30% after 12 h incubation for all the P407-tested concentrations (See Figure 3). The minimal release of RLX into the GIT pH conditions will ensure the higher bioavailability of RLX via NLCs. The un-released RLX from the NLCs can be absorbed via the lymphatic transport of NLCs that significantly enhances the bioavailability of RLX while also surpassing the Pgp efflux in the intestine.

### 3.5. Differential Scanning Calorimetry

**Figure 4:**
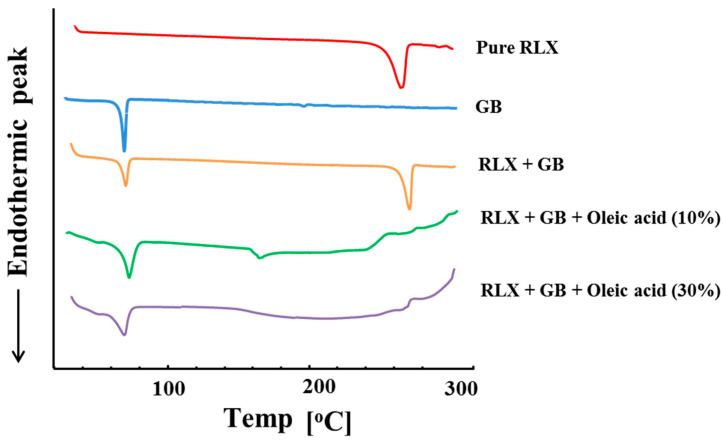
Differential scanning calorimetry (DSC) thermograms of the physical mixture of ingredients used in the NLCs. GB: glyceryl behenate; RLX: raloxifene hydrochloride.

The DSC thermogram of the formulation ingredients and the mixture of ingredients is shown in Figure 4. The sharp peak in the thermogram of RLX at 271 °C corresponds to the melting peak of the RLX crystals. The RLX peak remains unchanged in the physical mixture of glyceryl behenate and RLX. However, the endothermic peak height of RLX was reduced and shifted to 160 °C in the melt dispersion of lipid with 10% oleic acid and the sharp peak disappeared in the melt dispersion having 30% oleic acid. A broad dip in the baseline was seen in the mixture having 30% oleic acid. The shift of the peak and the peak broadening shows the change in the degree of crystallinity. Therefore, it can be concluded that the crystallinity of RLX was significantly reduced due to the dispersion of raloxifene in the lipid–oil matrix. The change in the degree of the crystallinity of glyceryl behenate can also be seen by comparing the heat of enthalpy per gram. The measured heat of enthalpy (in J/g) for pure glyceryl behenate, 10% oleic acid mixture and 30% oleic acid mixture was −75.89, −63.74 and −51.58, respectively. The decrease in the heat of enthalpy per gram of lipid shows a decrease in the crystallinity of the lipid in the presence of the oleic acid. Similarly, the peak broadening was also seen for glyceryl behenate in the melt dispersion which showed the reduction in crystallinity of glyceryl behenate. It can be attributed to the presence of oleic acid in the melt dispersion which helps in preventing the recrystallization of glyceryl behenate as well as RLX. Therefore, the shift of the peak and reduction in peak height whether in lipid or drug can be attributed to the matrix which was composed of the mixture of lipids.

### 3.6. Pharmacokinetics

**Figure 5:**
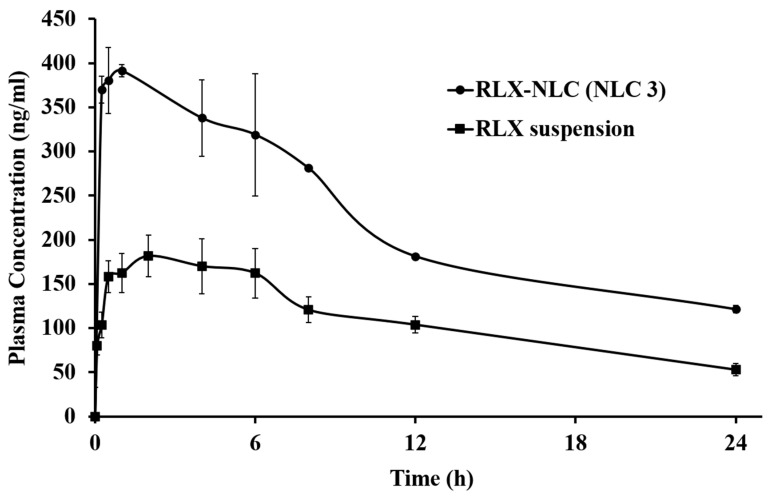
Pharmacokinetic profile of RLX for 24 h after oral administration of the optimized NLC formulation (NLC 3) and the RLX suspension in female Wistar rats. Data are given as the mean ± SD (*n* = 3).

The NLC 3 was carried forward for the in vivo study as it showed the optimum average size (lowest), PDI and zeta potential. The pharmacokinetic parameters of the RLX suspension, RLX-NLC (NLC 3) is presented in Table 4 and its pharmacokinetic profile in Figure 5. A significant enhancement in the oral bioavailability by 3.1-fold with RLX-NLC was observed as compared to the RLX suspension. This can be attributed to the low drug release in the GIT as supported by the in vitro drug release and absorption of both the free drug and NLCs via different absorption pathways [23,47]. The absorption pathways for the NLCs could be through the lymphatic uptake by M cells in the intestine. In the earlier study from our group, based on the uptake study of the SLN produced from glyceryl behenate, it was shown that clathrin- and caveolae-mediated endocytosis contributed to the uptake from rat intestine. Furthermore, the passive drug diffusion through the micellar absorption could also be contributing to the drug’s bioavailability enhancement. Since the cumulative drug release from the formulation in the pH 1.2 and pH 6.8 buffers was less than 30%, the lymphatic pathway could therefore be the major absorption route for the bioavailability enhancement of RLX-NLC. The peak plasma concentration for RLX-NLC was twice as much as that for the RLX suspension. The time to reach maximum plasma concentration was the same for the RLX suspension and the RLX-NLC but the mean residence time (MRT) for the NLCs was more than twice of that for the compared RLX suspension. Furthermore, the clearance of the RLX in rats given RLX suspension was higher than that of the rats given RLX-NLC. The decrease in clearance, increase in MRT, increase of plasma t_1/2_ and increase in plasma C_max_ supported the oral bioavailability enhancement and the potential for better therapeutic treatment. Recently, Hosny et al. reported the enhancement in the clinical bioavailability of RLX using NLCs [48].

### 3.7. Stability Studies

**Figure 6:**
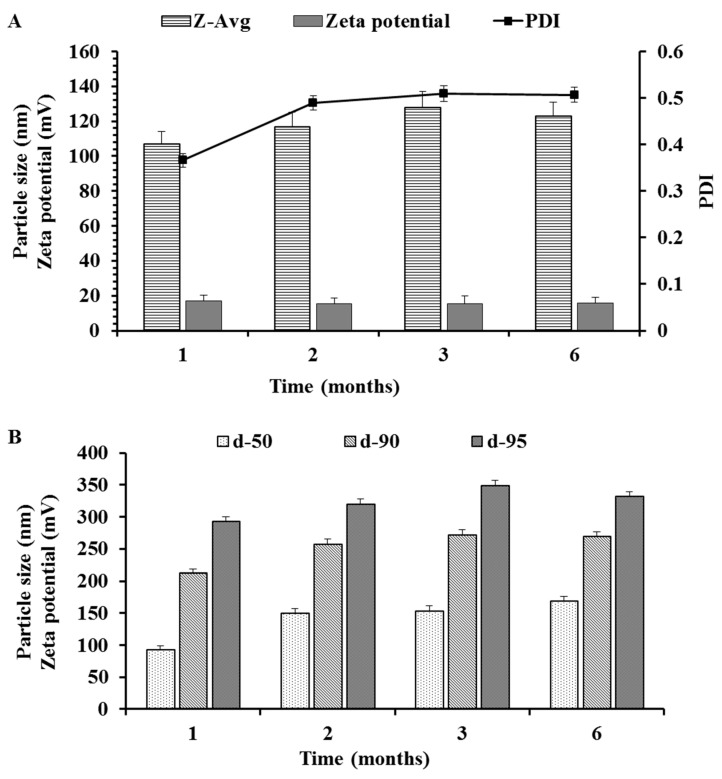
Stability of the particles as a function of time at 4 °C. (**A**) Particle size as the Z-average, polydispersity index (PDI) and zeta potential. (**B**) Particle sizes of d50, d90, d95.

**Figure 7:**
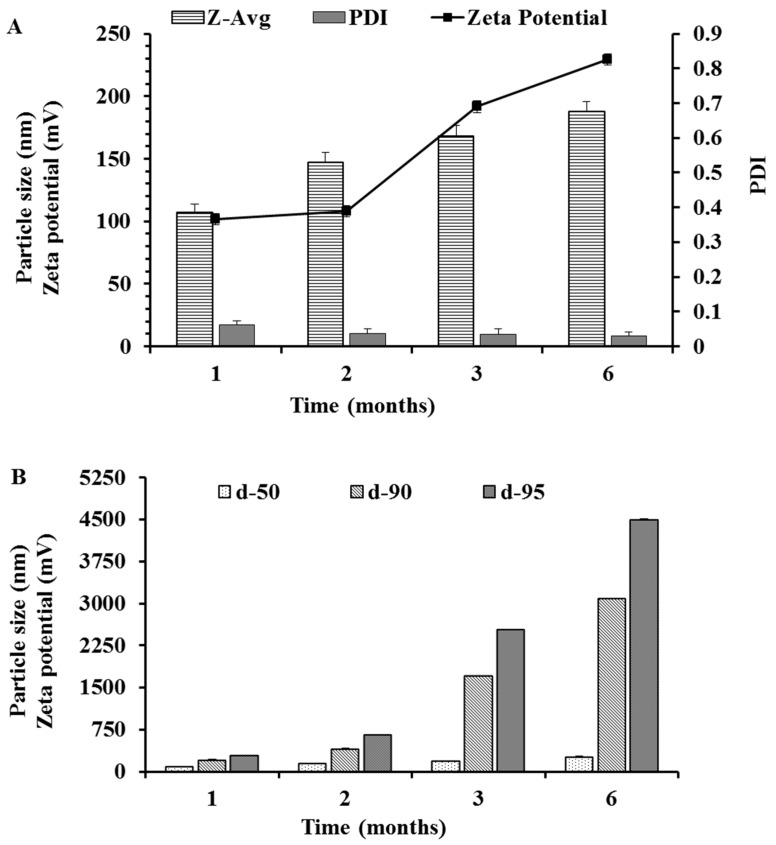
Stability of the particle as a function of time at room temperature. (**A**) Particle sizes as the Z-average, PDI and zeta potential. (**B**) Particle sizes of d50, d90, d95.

**Figure 8:**
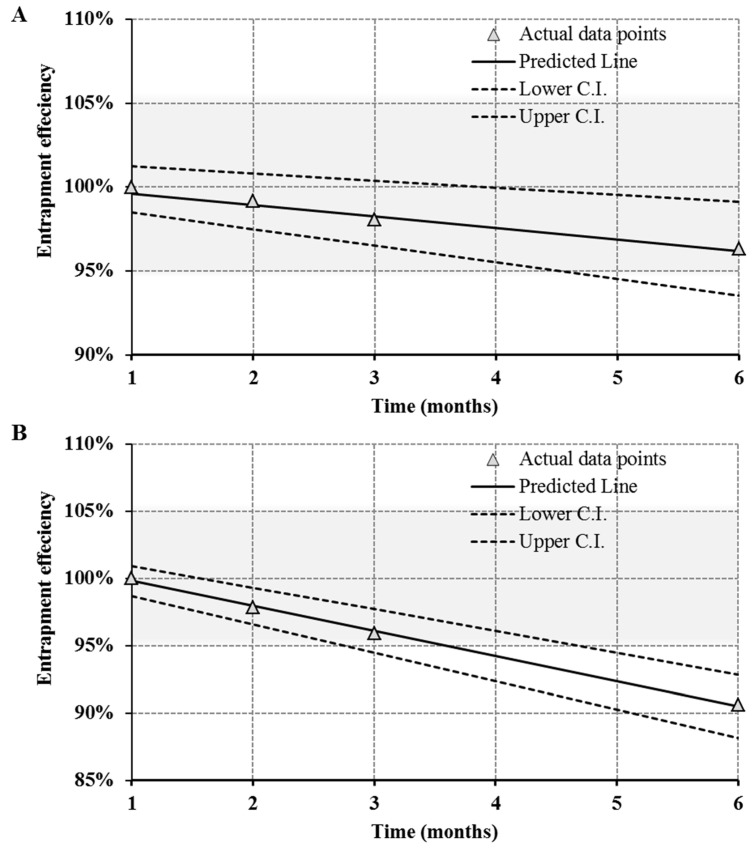
Shelf-life estimation of the RLX-solid lipid nanoparticles (SLNs) (**A**) at 4 °C and (**B**) at 25 °C ± 2 °C and 60% ± 5% RH. Dotted line shows the 95% confidence interval limits.

The optimized NLC formulation (i.e., NLC3) that had a balance of low size, narrow size distribution, enough zeta potential and good entrapment efficiency, was accessed for stability, and the same formulation was also studied for bioavailability enhancement (Figure 5). The stability was accessed based on changes in particle characteristics (Figure 6 and Figure 7) and changes in the % EE (Figure 8). The slight increase in PDI and particle size (Figure 6) while the slight decrease in zeta potential and % EE (Figure 8A) were observed over a 6-month storage period at 4 °C. However, significant changes were observed in NLCs stored at room temperature (25 °C ± 2 °C and 60% ± 5% RH) (Figure 7 and Figure 8B).

If only Z-average is recorded for stability, then a significant change in particle characteristics cannot be predicted because some percentage of bigger particles will not affect the Z-average. However, the difference can be easily seen when comparing other particle characteristics such as undersized particle sizes (d50, d90 and d95), zeta potential, PDI and % EE. The PDI, d90 and d95 (Figure 7) were significantly increased over the period of 6 months storage at room temperature while the % EE (Figure 8) and the zeta potential (Figure 7) were decreased. This can be attributed to the expulsion of the drug with the oozing of oleic acid that simultaneously causes a decrease in zeta potential, as well causing the aggregation of particles and an increase in the PDI. The effect of the storage conditions on the % EE is shown in Figure 8. The reduction in % EE was seen at both storage conditions, but it was lesser in the NLCs stored at 4 °C than NLCs stored at room temperature. The drug expulsion from the lipid nanocarriers upon ageing has been reported earlier, which is due to the crystallization of the solid lipid matrix causing the expulsion of the drug subsequently leading to a reduction in the % EE [49,50]. Therefore, to estimate the shelf-life of the optimized NLCs, the time taken to reach 90% of initial % EE (t90_EE_) was considered as a stability indicator. As predicted and shown in Figure 8, the % EE of the NLCs stored at 4 °C after 6 months ranged (at 95% confidence interval (C.I.)) from 99.11% (upper C.I.) to 93.5% (lower C.I.), with a mean of 96.18% while for NLCs stored at room temperature the % EE ranged from 92.87% (upper C.I.) to 88.16% (lower C.I.), with a mean of 90.51%. The NLCs being more stable at 4 °C can be attributed to the reduced expulsion of the drug because of the changes that occurred in the crystal lattice of the glyceryl behenate. In addition, the oleic acid oozing out would be least at 4 °C because the storage temperature was significantly below the congealing temperature of the oleic acid. In addition, the presence of oleic acid also prevents the expulsion of RLX. Therefore, we suggest the storage of NLC at 4 °C for better stability.

## 4. Conclusions

The nanosized RLX-loaded NLCs were prepared in the study using a combination of hot homogenization and sonication, with a high (>90%) encapsulation efficiency. The calorimetric study showed a reduction in the crystallinity of RLX in the dispersion of lipid–oil. The processing parameters like the amount of oleic acid, sonication time, homogenization and P407 concentration, can all affect the NLC characteristics including the particle size, zeta potential, % EE, % CR and the NLC stability. The RLX-NLC (120 nm average size, +14 zeta potential) based on glyceryl behenate and oleic acid showed potential to enhance the oral bioavailability of RLX in rats. RLX-NLC also improved the pharmacokinetic parameters of RLX. The RLX-NLC were physically stable and retained the RLX encapsulation (>90% from initial % EE) at cold conditions for over 6 months of storage. Overall, the RLX-NLC can be a potential therapeutic delivery strategy in osteoporosis while also providing a better shelf life.

## Figures and Tables

**Table 1:** Composition and the variables (HT-homogenization time, ST-sonication time) of various nanostructured lipid carrier (NLC) formulations for optimization. The % P407 refers to the concentration used to prepare the NLCs while the final % P407 will be lesser after the dilution to 50 mL.

Code	Drug (mg)	Glyceryl Behenate (g)	Oleic Acid (g)	P 407 (% *w/v*, [mL])	Water q.s. (mL)	HT (min)	ST (min)
NLC 1	NA	0.7	0.3	5.0 [20]	50	3	10
NLC 2	50	0.7	0.3	5.0 [20]	50	NA	10
NLC 3	50	0.7	0.3	5.0 [20]	50	3	10
NLC 4	50	0.7	0.3	5.0 [20]	50	3	20
NLC 5	50	0.7	0.3	2.5 [20]	50	3	10
NLC 6	50	0.7	0.3	7.5 [20]	50	3	10
NLC 7	50	0.77	0.23	5.0 [20]	50	3	10
NLC 8	50	0.83	0.17	5.0 [20]	50	3	10
NLC 9	50	0.89	0.09	5.0 [20]	50	3	10

**Table 2:** Particle characteristics of the various NLC formulations for optimization.

Code	d-avg (d.nm)	PDI	d50 (d.nm)	d90 (d.nm)	d95 (d.nm)	Zeta Potential (mV)
NLC 1	124	0.191	115	227	267	−13.2
NLC 2	310	0.233	280	521	621	+8.71
NLC 3	120	0.293	97	209	270	+14.4
NLC 4	142	0.259	123	250	302	+12.3
NLC 5	132	0.224	124	240	288	+14.8
NLC 6	179	0.305	120	271	329	+11.7
NLC 7	115	0.380	173	492	659	+15.2
NLC 8	182	0.319	255	572	689	+17.8
NLC 9	247	0.383	342	790	1100	+15.4

**Table 3:** In vitro raloxifene hydrochloride (RLX) release and EE (%) of the selected NLC formulations. NLC 3, 5 and 6 are with varied surfactant concentrations while NLC 4 is with varied sonication time. EE (%)—percentage entrapment efficiency; CR (%)—percentage cumulative release.

Code	EE (%)	Free Drug (%)	CR (%)	CR (%, Excluding Free Drug)
**NLC 3 (5% P407)**	91.71	8.29	28.48	20.19
**NLC 6 (7.5% P407)**	88.13	11.87	30.16	18.29
**NLC 5 (2.5% P407)**	94.51	5.49	18.68	13.19
**NLC 4 (5% P407)**	92.07	7.93	30.71	22.79

**Table 4:** Pharmacokinetic parameters of RLX in the oral administration of the optimized NLC formulation (NLC 3) and the RLX suspension in female Wistar rats. * *p* < 0.05.

Parameter	RLX Suspension	RLX-NLC
**C_max_ (ng/mL)**	181.71 ± 17.83	391.35 ± 32.53 *
**T_max_ (h)**	2.00 ± 0.32	1.00 ± 0.25 *
**MRT (h)**	13.00 ± 1.17	19.09 ± 1.05 *
**AUC_(0-t)_ (µg.h/mL)**	2.49 ± 0.23	7.71 ± 0.34 *
**t_1/2_ (h)**	13.21 ± 1.35	20.00 ± 2.13 *
**F_rel_**	-	3.19

MRT: Mean residence time; AUC_(0-t)_: Area under the curve between 0 to time ‘t’.
